# Supplementary material for: High-dimensional cortical signals reveal rich bimodal and working memory-like representations among S1 neuron populations
Source: Commun Biol. 2024 Aug 23;7:1043. doi: 10.1038/s42003-024-06743-z (PMC11344095; doi:10.1038/s42003-024-06743-z)
Supplement: Supplementary file 2 — Supplementary Information [file 42003_2024_6743_MOESM2_ESM.pdf]

Supplementary Figures

For the paper: High-dimensional cortical signals reveal rich bimodal and working memory-like representations among S1 neuron populations.

By Sofie S. Kristensen, Kaan Kesgin, Henrik Jörntell

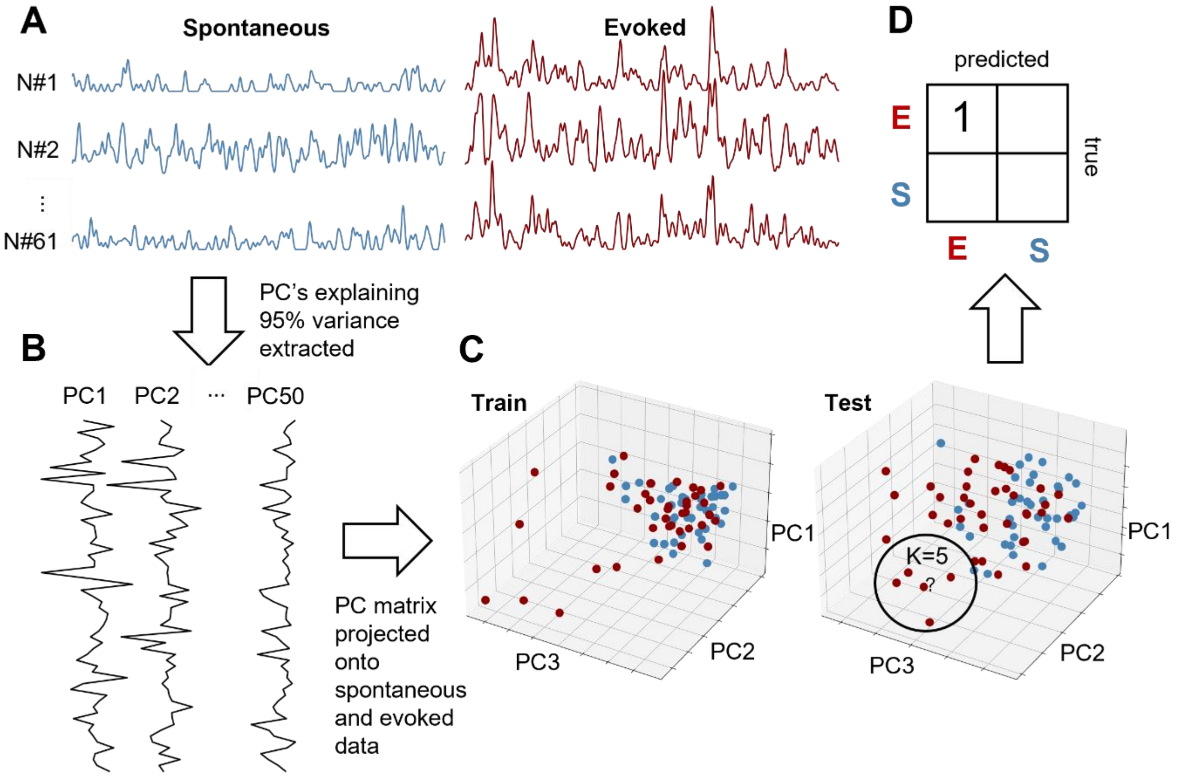

Supplementary Figure 1: Illustration of the PCA+kNN approach. A) Time continuous vectors of spontaneous activity (blue) and tactile evoked activity (red) from three example neurons. B) Three example principal components derived from the full spontaneous data set. PCs explaining 95% of the variance resulted in 50 PCs with the length 61 (equivalent to the number of neurons in this example experiment). C) Example visualization of the placement of data points from the spontaneous data (blue) and the tactile evoked data (red) in a three-dimensional PC space, after splitting all the data into a training set and a test set. D) Example result of the kNN estimation for one data point (question mark in C) after training.

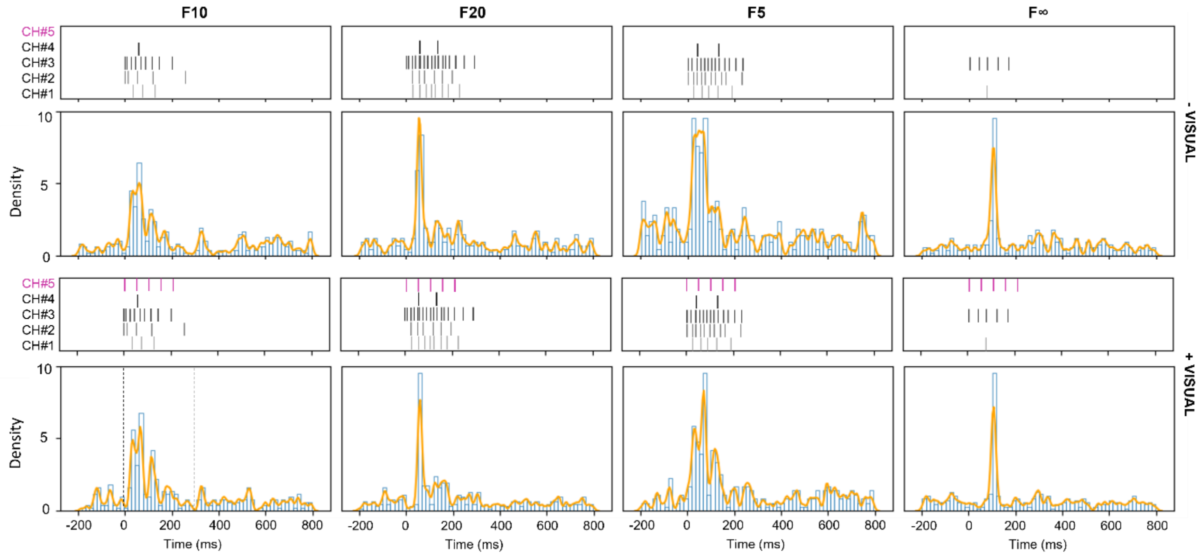

Supplementary Figure 2: Illustration of the stimulus combinations (the 4 tactile stimulation patterns with or without the visual stimulus) and responses from a sample S1 neuron. Top row: Illustration of the four spatiotemporal activation patterns without visual flashes and below PSTH with KDE. Bottom row: Stimulations patterns with visual flashes and below PSTH and KDE. The dashed lines illustrate the time window analyzed in the neuron-by-neuron PCA+kNN analysis. It can be noted that comparisons between the four columns of histograms are suggestive of some potential differences, but these differences were not strong enough to be statistically significant. However, such differences became more systematic when we instead focused our analysis to the activity distribution patterns across the S1 neuron populations.

Supplementary Table

		PCs explaining 95% / PCs explaining 100%					
**Expt.#**	**Neurons**	**1 ms**	**5 ms**	**10 ms**	**50 ms**	**100 ms**	**300 ms**
1	40	27/40	18/40	12/40	5/38	3/35	2/13
2	62	50/62	40/62	30/62	8/62	5/42	2/13
3	70	55/70	40/70	28/70	7/44	4/22	2/6
4	66	42/66	31/66	26/66	11/61	7/61	3/28
5	36	28/36	24/36	20/36	8/36	5/33	2/13

Supplementary Table 1: PCs explaining 95% and 100% of the variance in the spontaneous covariance patterns of all experiments when data is down-sampled into different bin sizes.
